# Integrating mental health assessment and intervention into cleft care: a prospective cohort study from a tertiary clinic in India

**DOI:** 10.1186/s13034-026-01040-5

**Published:** 2026-02-13

**Authors:** Ajay Aditya Aadhi Mani, Aksha Gaunekar

**Affiliations:** Department of Psychiatry, Institute of Psychiatry and Human Behaviour, Goa Medical College, Bambolim, Goa 403202 India

## Abstract

**Background:**

CLP is a complex congenital anomaly with considerable psychosocial and medical implications. Despite recognised psychiatric co morbidity, mental health integration in cleft services remains uncommon in LMICs. This study prospectively evaluated a psychiatry-integrated multidisciplinary cleft clinic in a public tertiary hospital in India.

**Methods:**

Consecutive patients aged ≤ 18 years attending a psychiatry-integrated multidisciplinary cleft clinic at Goa Medical College (January 2022–September 2024) were prospectively followed for 12 months. Psychiatric screening was conducted using the Strengths and Difficulties Questionnaire, followed by disorder-specific assessments where indicated, with diagnoses formulated according to ICD-10 criteria. Follow-up continuity was categorised as regular, irregular, or dropout (≥ 90-day lapse). Feasibility and acceptability of the integrated model were assessed using predefined service-delivery indicators and structured caregiver feedback.

**Results:**

Of 224 registered patients, 210 met the inclusion criteria (mean age 9.8 ± 4.3 years; 56.2% male). Regular, irregular, and dropout follow-up rates were 45.7%, 25.7%, and 28.6%, respectively. Psychiatric comorbidity was identified in 53.3% of participants and was significantly associated with follow-up status (*p* = 0.009), with a higher prevalence among irregular attendees and dropouts. Better follow-up continuity was significantly associated with younger age, higher maternal education, higher socioeconomic status, urban residence, and completion of planned surgical milestones. Speech therapy involvement differed significantly across follow-up groups (*p* = 0.002), with higher engagement among irregular attendees and dropouts, whereas hearing impairment showed no association with follow-up (*p* = 0.48).From a feasibility perspective, 93.8% of registered patients were enrolled, baseline psychiatric assessment was completed for all participants, and all children with identified psychiatric morbidity received at least one mental health intervention. Caregiver feedback (n = 150) indicated high satisfaction with integrated care and perceived stigma reduction; dissatisfaction was infrequent (11.3%) and primarily related to logistical barriers.

**Conclusions:**

Embedding mental health services within multidisciplinary cleft care is feasible and acceptable in LMIC settings and is associated with improved engagement and continuity of care. Structured psychosocial assessment and coordinated interventions may help address psychiatric morbidity and support sustained participation in cleft care.

**Supplementary Information:**

The online version contains supplementary material available at 10.1186/s13034-026-01040-5.

## Introduction

Cleft lip and palate (CLP) are among the most common congenital anomalies worldwide, with an incidence of 1 in 600–800 live births (1.42 per 1,000). Isolated cleft palate occurs less frequently, with approximately 1 in 2,000 live births. Cleft lip alone accounts for 15% of cases, cleft lip with palate accounts for 45%, and isolated cleft palate accounts for 40%. CLP results from the failure of fusion of the maxillary and medial nasal processes during early embryogenesis [1]. In addition to structural deformities, CLP affects feeding, speech, hearing, dentition, and growth. The children face stigma, bullying, and peer rejection due to facial differences and speech impairment, leading to low self-esteem and social withdrawal. Parents frequently report guilt, heightened caregiving burdens, and distress, making CLP both a medical and psychosocial condition requiring multidisciplinary care [2]. Despite surgical and rehabilitative advances, the broader psychological impact often persists [3, 4].

Psychiatric comorbidities are increasingly recognised in CLP patients. Anxiety, depression, social withdrawal, attention-deficit hyperactivity disorder, and learning difficulties are frequently reported. Adolescents are especially prone to body image concerns, social anxiety, and poor quality of life. Impaired speech intelligibility and hearing loss further compound social exclusion and negative self-perception [4, 5].

Large population-based studies show that children with cleft lip and/or palate (CLP) have higher rates of mental health and behavioural disorders than age- and sex-matched peers. A national database study by Lorenz et al. reported increased prevalence of anxiety, depressive disorders, attention-deficit/hyperactivity disorder, and disruptive behavioural conditions, with greater psychotropic medication use, particularly among children with palatal involvement and associated hearing morbidity [6]. Evidence from low- and middle-income countries remains limited; however, an Indian hospital-based study by Avinash et al. similarly identified substantial psychosocial and behavioural difficulties, albeit with methodological constraints. Collectively, the literature suggests that while many children with CLP adapt adequately, clinically significant subgroup experiences psychiatric and selected neurodevelopmental morbidity. The impact of these comorbidities on follow-up engagement and continuity of care in routine multidisciplinary settings remains poorly characterised [7].

Integrated care models embedding mental health services within paediatric speciality clinics are associated with improved engagement and follow-up. Co-located psychiatric care reduces access barriers and stigma, enabling earlier identification of psychosocial difficulties [8]**.** Evidence from paediatric collaborative-care settings demonstrates better clinic attendance and reduced attrition, particularly for children requiring longitudinal care [9].

However, mental health assessments and interventions remain under prioritised across low- and middle-income countries (LMICs), including India, where surgical and speech outcomes dominate the clinical focus [10–12]. Longitudinal studies such as that of Nicholls et al. have shown that adults with CLP continue to experience low self-esteem, social isolation, and higher psychiatric morbidity than their peers do, underscoring the need for early, sustained mental health input [13].

International guidelines advocate multidisciplinary cleft care involving surgeons, orthodontists, prosthodontists, speech therapists, audiologists, and social workers [14]. In high-income contexts, psychologists and psychiatrists are integral to such teams, conducting routine screening, early intervention, and longitudinal support [3]. The evidence suggests that timely mental health integration enhances adherence, reduces dropout, and improves long-term psychosocial outcomes [4]. In contrast, LMIC cleft services rarely include psychiatric input. Limited manpower and poorly defined referral pathways lead to unrecognised morbidity and delayed help-seeking. Even surgical needs remain unmet—recent modelling from 113 countries estimated over 600,000 unrepaired cases, with the highest burden on fragile health systems such as Cambodia, Afghanistan, and Nepal [15]. When surgical care is so underserved, psychosocial needs are inevitably neglected.

The present study aims to generate such evidence by prospectively evaluating the integration of psychiatry within a multidisciplinary cleft-palate clinic at Goa Medical College. Specifically, it examines the following:

1. The prevalence and profile of psychiatric comorbidities identified in children and adolescents with CLP.
2. The associations between psychiatric morbidity and follow-up continuity, including attendance and dropout, were explored.
3. The feasibility and utility of an integrated psychiatry model within a public-sector, resource-constrained setting.

To achieve this goal, consecutive CLP patients were followed for 12 months to document psychiatric morbidity, adherence, and service outcomes in a psychiatry-augmented multidisciplinary care framework.

## Materials and methods

This prospective cohort study was conducted at the Cleft Lip and Palate Clinic, Goa Medical College, a tertiary-care government facility in western India. This study evaluated the integration of psychiatry within a multidisciplinary cleft care model and its impact on psychiatric comorbidities and follow-up outcomes. All children and adolescents (≤ 18 years) who registered at the cleft clinic during the study period (January 2022–September 2024) were eligible for enrollment. Consecutive participants were enrolled at their initial clinic visit (baseline) and prospectively followed for 12 months. Clinical, psychiatric, and service-related data were recorded at each clinic visit during this observation window. For analysis, follow-up continuity was categorised as regular, irregular, or dropout, according to the predefined 90-day threshold.

### Inclusion criteria

1. Children and adolescents (≤ 18 years) with a diagnosis of cleft lip and/or palate (CLP) were confirmed by the surgical team.
2. Provided informed consent/assent and agreed to scheduled psychiatric evaluations and follow-up assessments over 12 months.

### Exclusion criteria

1. Syndromic clefts associated with severe neurological or developmental impairment precluding reliable psychiatric evaluation.
2. Children with non-CLP craniofacial anomalies identified via confirmatory examination.
3. Families who declined consent/assent for psychiatric assessment or longitudinal follow-up.
4. Patients who were transferred care or were lost to follow-up before baseline psychiatric evaluation.

### Study setting

The Cleft Lip and Palate Clinic at Goa Medical College provided team-based care, including plastic surgery, orthodontics, prosthodontics, dentistry, speech and language therapy, and psychiatry. The psychiatry unit was integrated into the cleft team. Participants received routine psychosocial screening at baseline, parent guidance and psychoeducation, diagnosis and management of psychiatric comorbidities, and counselling on treatment adherence. In addition, brief counselling sessions addressed stigma reduction, coping with bullying and social anxiety, and caregiver support, to enhance long-term psychosocial adjustment.

All services were provided free of cost under the government-run system. Follow-up visits were scheduled according to multidisciplinary needs, and psychiatric medications, if prescribed, were dispensed from the hospital pharmacy for 15–60 days.

### Enrollment and follow-up schedule

Consecutive eligible participants were enrolled at their initial clinic registration (baseline, T0) and prospectively followed for a period of 12 months. Data were recorded at each clinic visit during this period. For reference, the key assessment points were T0 (baseline), T1 (approximately 3 months), T2 (6 months), and T3 (12 months), although visits outside these intervals were also captured as part of routine care. If in-person visits were missed, phone or tele-consult follow-up was permitted.

Retention procedures included reminder calls/SMS before appointments, documentation of travel-related issues, and bundling of multidisciplinary consultations on the same day whenever feasible.

### Data collection procedure

Data were collected prospectively via a structured case report form (CRF) and entered into Excel. At each time point, the following information was recorded: sociodemographic details (age, sex, education, family type and socioeconomic status); cleft-related variables (type of cleft, surgery status and speech therapy involvement); psychiatric assessment findings (screening, diagnoses, comorbidities, and interventions provided); and follow-up data (clinical attendance, dropout, and continuity of care). Source documents included clinician notes and clinic registers, and all entries were time-stamped.

### Follow-up categorisation and defining dropouts

Patients were categorised according to follow-up status within the first 12 months post-enrollment to ensure a uniform observation window:

1. Regular attendees—those who maintained scheduled follow-up with no lapse exceeding 90 days.
2. Irregular attendees—those with at least one lapse in follow-up exceeding 90 days who subsequently re-engaged with services within the 12 months.
3. Dropouts—those who failed to return for follow-up within 90 days of their last documented visit during the 12 months.

The 90-day threshold reflected the maximum duration of medication or therapy dispensation at the study site (15–60 days) and approximated one to two missed refills or therapy cycles, making it a commonly used benchmark in outpatient adherence studies to indicate disengagement from care.

### Age categorisation

For analysis, participants were stratified into three developmental age groups, which were consistent with categories commonly used in child psychiatry and paediatrics: early childhood (0–6 years), middle childhood (7–12 years), and adolescence (13–18 years). This classification enabled comparisons of clinical and follow-up patterns across developmental stages.

### Parental employment status

Parental employment status was coded as “employed” if at least one parent was engaged in any form of paid work, including salaried employment, self-employment, or informal/daily wage labour. Families were coded as “unemployed” if neither parent reported paid work of any kind. This broad categorisation was intended to capture the household’s economic resource base rather than the stability or formality of employment.

### Psychiatric comorbidity assessment

All children attending the Cleft Lip and Palate Clinic underwent psychiatric evaluation by a consultant psychiatrist as part of routine multidisciplinary care. Diagnostic formulation followed the International Classification of Diseases, 10th Revision (ICD-10) clinical guidelines, with age-appropriate adaptations. The assessment was conducted in three steps:

#### Initial screening

All participants were screened using the Strengths and Difficulties Questionnaire (SDQ)—parent-rated for all children, with self-reports for adolescents where feasible. The SDQ provided a broad profile of emotional, behavioural, hyperactivity, and peer-related domains, enabling early detection of psychosocial concerns.

#### Targeted assessment for positive findings or clinical concerns

Children who screened positive on the SDQ or in whom clinical interviews indicated difficulties underwent disorder-specific or age-specific evaluations, including:

- *Hamilton Anxiety Rating Scale* (HAM-A) for anxiety symptoms
- *Hamilton Depression Rating Scale* (HAM-D) for depressive symptoms
- *Vanderbilt ADHD Diagnostic Rating Scale* (parent/teacher forms) for ADHD
- *NIMHANS Specific Learning Disability Battery*, supplemented by school history, for SLD
- *Developmental Assessment Scale for Indian Infants* (DASII) for children aged 0–3 years, assessing developmental and socio-emotional functioning through standardised Indian norms
- Detailed clinical assessment for adjustment disorders and other behavioural or stress-related conditions.

#### Final diagnosis

ICD-10 clinical criteria were applied, integrating findings from clinical interviews, standardised rating scales, collateral history, and school records. For younger children, emphasis was placed on developmental and behavioural indicators derived from caregiver reports and clinical observation, ensuring age-appropriate formulation.

Autism spectrum disorder was not analysed as a predefined diagnostic outcome in this study. Children with suspected ASD were managed clinically within the multidisciplinary team; however, ASD was not coded as a separate analytic diagnostic category, as the protocol focused on psychiatric and behavioural conditions with immediate relevance to clinic engagement and follow-up. For analytic purposes, psychiatric diagnoses were grouped into six categories:

1. Anxiety disorders
2. Depressive disorders
3. Attention-deficit/hyperactivity disorder (ADHD)
4. Specific learning disorders (SLD)
5. Adjustment disorders
6. Other behavioural or stress-related conditions

For infants and preschool-age children (0–6 years), developmental or socio-emotional delays identified on the Developmental Assessment Scale for Indian Infants (DASII) were classified under ‘other behavioural/stress-related conditions’ for analytic purposes.

### Measures

#### Sociodemographic and clinical data

A semi-structured proforma was used to record age, sex, type of cleft anomaly, surgical history, developmental milestones, family characteristics, and socioeconomic status. Clinical information on surgery, speech therapy, dental, and ENT follow-up was obtained from multidisciplinary clinic records.

#### Child mental health assessment

Child emotional and behavioural difficulties were screened using the Strengths and Difficulties Questionnaire (SDQ), a widely used screening instrument with demonstrated reliability and validity for assessing emotional and behavioural domains in children and adolescents [16]. Developmental functioning in infants and preschool-aged children (0–6 years) was assessed using the Developmental Assessment Scale for Indian Infants (DASII). The DASII is a standardised, clinician-administered tool normed on Indian populations, which provides developmental quotients across motor and mental domains [17]. Findings were used to identify developmental or socio-emotional delays relevant to psychiatric formulation.

#### Caregiver mental health and stress

Caregiver symptoms of depression and anxiety were assessed using the Patient Health Questionnaire-9 (PHQ-9) [18] and the Generalised Anxiety Disorder-7 (GAD-7) scales [19], which are brief self-report measures with established reliability and validity in clinical settings. Caregiver perceived stress was assessed using the Perceived Stress Scale-10 (PSS-10) [20], a widely used instrument for assessing perceived stress over the preceding month. Standard cut-off scores were used to categorise symptom severity.

#### Clinical interview and diagnostic assessment

Children screening positive or referred by the multidisciplinary team underwent a psychiatrist-led clinical evaluation. Psychiatric diagnoses were made according to ICD-10 clinical criteria, and management plans were documented in standardised case records.

### Feasibility and acceptability measures

The feasibility and acceptability of the integrated mental health model were evaluated using predefined process indicators over the 12-month follow-up period. Feasibility was operationalised as the ability to enrol eligible children, complete baseline mental health assessments, and deliver at least one mental health intervention within routine multidisciplinary cleft-care services.

Acceptability was assessed through patterns of follow-up engagement, including attendance at scheduled in-person visits and continued engagement through telephone or tele-consult follow-up when in-person attendance was not feasible. Complete loss to follow-up was defined as failure to attend scheduled in-person visits and inability to establish telephonic contact during the follow-up period.

Feasibility indicators included enrollment rate, baseline assessment completion rate, and intervention delivery rate. Acceptability was examined descriptively using follow-up modality (in-person only, hybrid in-person and telephonic follow-up, tele-consultation only, or complete loss to follow-up). All indicators were summarised descriptively as counts and proportions.

### Family feedback

A structured caregiver feedback form was developed by the multidisciplinary cleft psychiatry team specifically for service evaluation. Items were selected a priori to reflect domains considered clinically relevant to integrated care delivery, based on routine clinic objectives and common concerns raised by families during consultations, including overall satisfaction with integrated care, perceived stigma reduction, adherence support, usefulness of psychiatric input, and caregiver stress relief. Closed-ended items were rated using simple categorical response options (e.g., satisfied/neutral/dissatisfied; yes/no) to maximise feasibility and comprehension across educational backgrounds, while open-ended sections allowed caregivers to elaborate on positive experiences or challenges.

Completed forms were collected at the end of clinic visits and archived with case records, yielding 150 analysable responses (approximately 71% response rate). Caregivers were permitted to endorse multiple viewpoints within a single form; therefore, feedback themes were not mutually exclusive. For analysis, closed-ended responses were coded as categorical variables. Open-ended responses were reviewed independently by two investigators and were analysed using a descriptive, inductive approach, with comments grouped into predefined service-related domains: satisfaction with integrated care, stigma reduction, adherence and follow-up engagement, school or peer-related support, caregiver stress relief, and dissatisfaction. Responses were classified as indicating dissatisfaction when caregivers described unmet expectations, logistical barriers (e.g., travel or time burden), or negative perceptions of psychiatric involvement.

### Statistical analysis

Statistical analysis was performed using SPSS version 29.0 (IBM Corp., Armonk, NY, USA). A two-tailed *p*-value of < 0.05 was considered statistically significant. Descriptive statistics were used to summarise the data. Continuous variables were expressed as means ± standard deviations (SD) or medians with interquartile ranges (IQR), depending on their distribution, while categorical variables were presented as frequencies and percentages.

Group differences across follow-up categories (regular, irregular, and dropout) were assessed using chi-square tests for categorical variables and one-way analysis of variance (ANOVA) or Kruskal–Wallis tests for continuous variables, as appropriate.

## Results

During the enrollment window (January 2022–September 2024), 224 children with cleft lip and/or palate were registered at the multidisciplinary clinic. Fourteen patients were excluded for predefined reasons: syndromic clefts with severe neurological impairment precluding psychiatric assessment (n = 5), declined consent/assent (n = 4), transfer of care before baseline psychiatric evaluation (n = 3), and non-CLP diagnoses on confirmatory examination (n = 2). The final analytic cohort comprised 210 participants. In total, 96 children (45.7%) met the criteria for regular follow-up, 54 (25.7%) met the criteria for irregular follow-up, and 60 (28.6%) met the criteria for dropout.

The mean age at enrollment was 9.8 years (SD = 4.3; range = 1–18 years). Age differed significantly across follow-up categories [F (2,207) = 4.3, *p* = 0.015]: regular attendees were younger (mean 8.7 years) than irregular attendees (mean 10.2 years) and dropouts (mean 11.1 years). When stratified by developmental stage, the 0–6 years group contributed the largest proportion of regular attendees, whereas dropouts showed a trend toward clustering in the 7–12 years group (χ^2^ = 9.29, *p* = 0.054).

The gender distribution did not vary significantly by follow-up status (χ^2^ = 0.25, df = 2, *p* = 0.88). Males constituted 56.2% of the overall cohort and were proportionally represented in each subgroup.

Residence was significantly associated with follow-up status (χ^2^ = 7.35, df = 2, *p* = 0.025): children from urban households were overrepresented among regular attendees, whereas rural domiciles were more common among dropouts. The broader domicile categories (within Goa, outside Goa and outside India) did not differ significantly across the follow-up groups (χ^2^ = 3.89, df = 4, *p* = 0.421).

Religious affiliation was not associated with follow-up status (χ^2^ = 0.17, df = 4, *p* = 0.996). Hindus accounted for two-thirds of the cohort, with Catholics and Muslims comprising 26.2% and 7.1% respectively, proportions that remained consistent across groups. Table 1 presents the sociodemographic characteristics of the participants.

**Table 1:** Sociodemographic details

Sociodemographic Variables	Entire cohort (*n* = 210), *n* (%)	Regular Attendees (*n* = 96), *n* (%)	Irregular Attendees (*n* = 54), *n* (%)	Dropouts (*n* = 60),* n* (%)	Test statistics (*P*)
Age means age (years)	9.8 ± 4.3	8.7 ± 4.0	10.2 ± 4.1	11.1 ± 3.7	*F* = 4.3
Range of age (years)	6 m – 18y	6 m -18 y	1.5y to 18y	8 m to 15.5 y	(0.015)
Age Distribution					*χ*^2^ = 9.29 (0.054)
0–6 years	72 (34.3)	40 (41.7)	16 (29.6)	16 (26.7)	
7–12 years	88 (41.9)	35 (36.5)	20 (37.0)	33 (55.0)	
13–18 years	50 (23.8)	21 (21.9)	18 (33.3)	11 (18.3)	
Gender					
Male	118 (56.2)	56 (58.3)	30 (55.6)	32 (53.3)	*χ*^2^ = 0.25 (0.88)
Female	92 (43.8)	40 (41.7)	24 (44.4)	28 (46.7)	
Address					
Urban	93 (44.3)	52 (54.2)	21 (38.9)	20 (33.3)	*χ*^2^ = 7.35 (0.025)
Rural	117 (55.7)	44 (45.8)	33 (61.1)	40 (66.7)	
Parental Education (mother)					
No education	45 (21.4)	10 (10.4)	13 (24.1)	22 (36.7)	*χ*^2^ = 29.76 (< 0.001)
Primary/middle school	68 (32.4)	26 (27.1)	20 (37.0)	22 (36.7)	
High school and above	90 (42.9)	57 (59.4)	17 (31.5)	16 (26.7)	
No mother	7 (3.3)	3 (3.1)	4 (7.4)	0(0%)	
Parental Education (Father)					
No education	40 (19.05)	15 (15.6)	12 (22.2)	13 (21.7)	*χ*^2^ = 5.81 (0.445)
Primary/middle school	70 (33.33)	30 (31.2)	20 (37.0)	20 (33.3)	
High school and above	95 (45.24)	49 (51.0)	22 (40.7)	24 (40.0)	
No father	5 (2.38)	2 (2.1)	0 (0.0)	3 (5.0)	
Socioeconomic status					
Lower socioeconomic status	95 (45.2)	30 (31.25)	28 (51.85)	37 (61.67)	*χ*^2^ = 26.33 (< 0.001)
Middle socioeconomic status	70 (33.3)	32 (33.3)	22 (40.74)	16 (26.67)	
High socioeconomic status	45 (21.4)	34 (35.42)	4 (7.41)	7 (11.66)	
Parental Employment status					
Employed	119 (56.67)	65 (67.71)	25 (46.30)	29 (48.33)	*χ*^2^ = 8.83 (0.012)
Unemployed	91 (43.33)	31 (32.29)	29 (53.70)	31 (51.67)	
Religion					
Hindu	140 (66.7)	63 (65.6)	36 (66.7)	41 (68.3)	χ^2^ = 0.17(0.996)
Catholics	55 (26.2)	26 (27.1)	14 (25.9)	15 (25.0)	
Muslim	15 (7.1)	7 (7.3)	4 (7.4)	4 (6.7)	
Domicile					
In Goa	152 (72.4)	65 (67.7)	40 (74.1)	47 (78.3)	χ^2^ = 3.89(0.421)
Outside Goa	42 (20.0)	21 (21.9)	10 (18.5)	11 (18.3)	
Outside India	16 (7.6)	10 (10.4)	4 (7.4)	2 (3.3)	

Maternal education was strongly associated with follow-up status (χ^2^ = 29.76, df = 6, *p* < 0.001). Nearly 60% of regular attendees had mothers educated at least at the high school level, whereas children of mothers with no formal education were disproportionately represented among dropouts. In contrast, paternal education was not significantly relatedto follow-up continuity (χ^2^ = 5.81, df = 6, *p* = 0.445).

Parental employment status was also significantly associated with attendance patterns (χ^2^ = 8.83, df = 2, *p* = 0.012). Children from households with at least one employed parent were more likely to remain in regular follow-up and less likely to disengage than those from unemployed households.

Socioeconomic status (SES) showed a graded relationship with follow-up continuity (χ^2^ = 26.33, df = 4, *p* < 0.001). Among regular attendees, 35.4% were from high-SES families, 33.3% were from middle-SES families, and 31.3% were from low-SESfamilies. In contrast, the dropouts were predominantly from lower socioeconomic backgrounds (61.7%), with only 11.7% from high-SES households.

From a feasibility perspective, of the 224 children registered at the multidisciplinary cleft clinic during the study period, 210 were enrolled into the psychiatry-integrated cohort (enrollment rate: 93.8%). Baseline mental health screening was completed for all enrolled participants (assessment completion rate: 100%). Among the 112 children identified with psychiatric comorbidity, all received at least one form of mental health intervention as part of routine multidisciplinary care, most commonly psychoeducation and parent guidance (intervention delivery rate: 100%). Table 2 summarises these feasibility indicators.

**Table 2:** Feasibility indicators of integrated mental health care

Feasibility domain	Definition	Value (%)
Enrollment rate	Children enrolled among all clinic registrations	210/224 (93.8)
Assessment completion rate	Completion of baseline mental health screening among enrolled children	210/210 (100)
Intervention delivery rate	Receipt of ≥ 1 mental health intervention among children with psychiatric comorbidity	112/112 (100)

Feasibility was operationalised using predefined process indicators reflecting reach, assessment delivery, and intervention delivery within routine multidisciplinary cleft care. Percentages are calculated using the appropriate denominators for each indicator

*At least one form of mental health input (most commonly psychoeducation and parent guidance) was delivered to all children diagnosed with psychiatric morbidity

Acceptability of the integrated mental health model was assessed through patterns of follow-up modality over the 12-month observation period. Of the 210 enrolled participants, 96 (45.7%) maintained regular in-person follow-up without requiring telephonic contact. A further 41 participants (19.5%) missed at least one scheduled in-person visit but re-engaged through a hybrid model combining in-person and telephonic follow-up, while 13 participants (6.2%) continued follow-up exclusively via tele-consultation after baseline assessment. Sixty participants (28.6%) were classified as complete dropouts, having missed in-person follow-up and being unreachable through telephonic contact. These patterns of follow-up engagement are summarised in Table 3.

**Table 3:** Follow-up modality as an indicator of acceptability

Follow-up modality	Definition	Value (%)
In-person follow-up only	Regular attendance without the need for tele/phone contact	96 (45.7)
Hybrid follow-up (in-person + tele/phone)	Missed ≥ 1 in-person visit but re-engaged via tele/phone	41 (19.52)
Tele-consultation only	Continued follow-up exclusively via tele/phone after baseline	13 (6.19)
Complete loss to follow-up	Missed visits and could not be contacted	60 (28.6)

Follow-up modality categories are mutually exclusive. Percentages are calculated using the total enrolled cohort (n = 210)

Surgical milestones were another key determinant of follow-up. Within 12 months, 147 patients (70.0%) underwent corrective surgery, whereas 63 (30.0%) were pending, primarily due to their young age at registration, the need for staged procedures, intercurrent medical conditions, or disengagement from services. Surgical completion rates differed significantly across follow-up categories (χ^2^ = 7.42, *p* = 0.024). Regular attendees had the highest completion rate (81.3%), followed by irregular attendees (68.5%) and dropouts (53.3%).

Post hoc comparisons indicated that dropouts were significantly less likely than regular attendees were to complete surgery (*p* = 0.018). In contrast, differences between irregular attendees and the other groups were not statistically significant. These findings suggested that failure to achieve surgical milestones may contribute to disengagement from multidisciplinary care. Table 4 shows the cleft-related characteristics, including the type of cleft, surgery status, and associated conditions.

**Table 4:** Cleft-related characteristics

Variables	Entire cohort (*n* = 210), *n* (%)	Regular attendees (*n* = 96), *n* (%)	Irregular attendees (*n* = 54), *n* (%)	Dropouts (*n* = 60),* n* (%)	Test statistics (*P*)
Type of cleft					*χ*^2^ = 2.87 (0.58)
Cleft lip only	65 (31.0)	30 (31.3)	16 (29.6)	19 (31.7)	
Cleft lip + palate	95 (45.2)	43 (44.8)	25 (46.3)	27 (45.0)	
Isolated cleft palate	50 (23.8)	23 (24.0)	13 (24.1)	14 (23.3)	
Surgery status					
Completed	147 (70.0)	78 (81.3)	37 (68.5)	32 (53.3)	*χ*^2^ = 7.42 (0.024)
Pending	63 (30.0)	18 (18.7)	17 (31.5)	28 (46.7)	
Hearing impairment					
Yes	116 (55.2)	54 (56.3)	33 (61.1)	29 (48.3)	*χ*^2^ = 1.48 (0.48)
No	94 (44.8)	42 (43.7)	21 (38.9)	31 (51.7)	
Speech therapy involvement (within 12 months)					
Yes	138 (65.7)	48 (50.0)	46 (85.2)	44 (73.3)	*χ*^2^ = 12.84 (0.002)
No	72 (34.3)	48 (50.0)	8 (14.8)	16 (26.7)	

Speech therapy involvement was documented in 138 patients (65.7%) within the 12-month observation period. Engagement was highest among irregular attendees (85.2%), followed by dropouts (73.3%) and regular attendees (50.0%), with a statistically significant difference across groups (χ^2^ = 12.84, *p* = 0.002).

Hearing impairment was identified in 116 patients (55.2%) and did not differ significantly across follow-up categories (χ^2^ = 1.48, *p* = 0.48). The prevalence of hearing difficulties was comparable across regular (56.3%), irregular (61.1%), and dropout (48.3%) groups, indicating no significant association with follow-up status. Table 5 summarises the distribution of psychiatric comorbidities among participants.

**Table 5:** Psychiatric Comorbidities

Variables	Entire cohort (*n* = 210), *n* (%)	Regular Attendees (*n* = 96), *n* (%)	Irregular Attendees (*n* = 54), *n* (%)	Dropouts (*n* = 60),* n* (%)	Test statistics (*P*)
*Overall status*					*χ*^2^ = 9.45 (0.009)
Any psychiatric comorbidity	112 (53.3)	41 (42.7)	32 (59.3)	39 (65.0)	
No psychiatric disorder	98 (46.7)	55 (57.3)	22 (40.7)	21 (35.0)	
*Specific diagnoses*					
Anxiety disorders	36 (17.1)	10(10.4)	12 (22.2)	14 (23.3)	
Depressive disorders	18 (8.6)	5 (5.2)	5 (9.3)	8 (13.3)	
ADHD	14 (6.7)	6 (6.3)	4 (7.4)	4 (6.7)	
Specificlearning disorder	20 (9.5)	7 (7.3)	5 (9.3)	8 (13.3)	
Adjustment disorders	24 (11.4)	8 (8.3)	7 (13.0)	9 (15.0)	
Other (behavioural/stress-related)	23 (10.9)	8 (8.3)	7 (13.0)	8 (13.3)	

Children could have more than one psychiatric diagnosis; therefore, subcategory totals exceed the overall “any psychiatric comorbidity” figure

Psychiatric disorders were identified in 112 children (53.3%), whereas 98 (46.7%) had no diagnosable psychiatric condition. The presence of psychiatric comorbidity was significantly associated with follow-up status (χ^2^ = 9.45, *p* = 0.009), with a higher prevalence among irregular attendees (59.3%) and dropouts (65.0%) than among regular attendees (42.7%).

Among specific diagnoses, anxiety disorders were the most common (17.1%), followed by adjustment disorders (11.4%), specific learning disorders (9.5%), and depressive disorders (8.6%). ADHD (6.7%) and other behavioural or stress-related conditions (10.9%) were less frequent. Anxiety disorders were disproportionately represented among dropouts (23.3%) relative to regular attendees (10.4%), while depressive, learning, and adjustment disorders also appeared more frequent among dropouts, though without statistical significance.

When examined by developmental stage, psychiatric morbidity was higher in middle childhood (60.2%) and adolescence (62.0%) than in early childhood (38.9%). Anxiety and depressive disorders clustered in adolescence, while ADHD and learning disorders were more prevalent in middle childhood. Adjustment and behavioural problems were observed across all age groups. Table 6 presents the psychiatric interventions delivered as part of the integrated care model.

**Table 6:** Psychiatric Interventions Provided

Intervention type	n (%) of patients with psychiatric comorbidity (n = 112)	n (%) of entire cohort (n = 210)
Psychoeducation and parent guidance	92 (82.1)	92 (43.8)
Brief counselling/supportive therapy	58 (51.8)	58 (27.6)
Behavioural interventions (ADHD, disruptive behaviours)	22 (19.6)	22 (10.5)
Cognitive-behavioural techniques (for anxiety/depression)	18 (16.1)	18 (8.6)
Pharmacological treatment	46 (41.1)	46 (21.9)
Referral and interdisciplinary coordination	20 (17.9)	20 (9.5)

Categories are not mutually exclusive; individual patients may have received more than one intervention

Among the 112 patients with psychiatric comorbidities, psychoeducation and parent guidance were the most frequently delivered interventions (82.1%). More than half of the participants received counselling or supportive psychotherapy (51.8%), whereas approximately one in five received behavioural interventions (19.6%) or referrals for extended care (17.9%). Pharmacological treatment was required in 41.1% of the cases, most commonly for ADHD and depressive disorders. Table 7 displays family feedback on the integrated psychiatric care model.

**Table 7:** Family feedback on integrated psychiatric care

Feedback theme	n (%)	Example documentation (qualitative notes)
Satisfaction with the integrated approach	142 (94.7)	“It is helpful that all doctors are in one place.”
Reduced stigma, improved acceptance	64 (42.7)	“Counselling helped us explain to relatives that this is not the child’s fault.”
Improved adherence/clinic attendance	55 (36.7)	“We came back because the doctor explained why follow-up is important.”
Support for school/peer difficulties	40 (26.7)	“The team gave a letter for school; it helped with bullying.”
Parent stress relief/coping	74 (49.3)	“Talking to a psychiatrist made us feel lighter.”
Dissatisfaction/unmet expectations	17 (11.3)	Too many visits are difficult because of travel costs

n = 150 completed feedback forms (71% of the total cohort). Themes were not mutually exclusive; a single respondent could endorse both positive and negative aspects (e.g., satisfaction with integrated care but difficulty attending due to distance). Percentages are calculated from the total number of forms

Family feedback was obtained from 150 caregivers (71% response rate). Because multiple themes could be endorsed per respondent, totals exceed 100%. Overall, 142 caregivers (94.7%) expressed satisfaction with the integrated approach, citing benefits such as reduced stigma, improved adherence, and relief of caregiver stress. Qualitative comments frequently highlighted appreciation for psychoeducation and school-related support. A smaller proportion (17 caregivers; 11.3%) noted challenges related to travel distance, time, or expectations of rapid speech or cosmetic improvement. These concerns often co-occurred with otherwise positive feedback.

## Discussion

### Overview of the present study

Integrating child psychiatry into routine cleft care represented a pragmatic and scalable model for addressing the psychosocial needs of children with craniofacial anomalies in resource-limited contexts. This study prospectively documented the impact of such integration, revealing high psychiatric comorbidity, its association with clinic engagement, and the acceptability of embedded services to families, thereby addressing a major evidence gap in LMIC cleft care.

Analyses indicated that more than half of children experienced at least one psychiatric comorbidity, and psychiatric morbidity was more common among those who disengaged from services than among those who remained engaged. Families reported high satisfaction with the integration of psychiatry into cleft care, particularly valuing early identification, counselling, and coordinated management. Together, these findings provided robust prospective evidence on the psychosocial dimensions of cleft care in LMICs and demonstrated the feasibility and acceptability of embedded psychiatric services.

### Psychiatric comorbidities in children with cleft lip and palate

Population-based and clinic-based studies consistently demonstrated an elevated burden of psychiatric morbidity among children with CLP. While Tillmann et al. reported that approximately 20% of children with CLP received at least one psychiatric diagnosis in population-based cohorts [21] and Lorenz et al. demonstrated significantly elevated relative risks for multiple psychiatric disorders compared with controls [6]; 53.3% of children in the present clinic-based cohort met criteria for at least one psychiatric diagnosis. Taken together, these findings indicated that children with CLP consistently experienced an increased risk of psychiatric morbidity across study designs and care settings.

In the present cohort, anxiety disorders were the most frequent internalising diagnosis (17.1%), followed by adjustment disorders (11.4%) and depressive disorders (8.6%). This pattern was comparable to findings reported by Millard et al., who documented elevated anxiety and depressive symptoms among children with cleft conditions using symptom-based assessments, relative to general paediatric populations [22]. Notably, anxiety disorders were clustered among children who disengaged from follow-up, echoing prior evidence that internalising psychopathology may heighten the risk of treatment dropout [23]. Although depressive disorders were less prevalent, they remained clinically important, particularly during adolescence, consistent with reports linking cleft conditions to lowered self-esteem, peer difficulties, and overprotective parenting, which may contribute to anxiety and depressive symptomatology [7, 24].

Specific learning disorders were identified in 9.5% of children, a prevalence lower than earlier clinic-based reports, such as Richman et al**.**, who found reading disability in approximately 35% of children with cleft conditions overall [25]. This lower prevalence likely reflected differences in age distribution, diagnostic thresholds, and access to interventions. Children with specific learning disorders showed higher rates of follow-up disengagement, suggesting that educational difficulties may have contributed to reduced clinic attendance [25, 26].

ADHD was identified in 6.7% of children in the present cohort. Consistent with this, population-based register studies, including Tillman et al., have reported a significantly increased risk of ADHD among children with nonsyndromic orofacial clefts compared with matched controls [21].

While psychotic spectrum disorders were rare in this predominantly paediatric cohort, this is developmentally expected. However, congenital anomaly-associated genetic syndromes such as 22q11.2 deletion syndrome confer markedly elevated lifetime risks of psychotic disorders [27]. These findings underscore the need for integrated cleft–mental health models to extend beyond common childhood comorbidities and support proactive, longitudinal monitoring for severe, late-onset psychiatric outcomes in high-risk subgroups.

### Interventions delivered

Nearly half of children with psychiatric morbidity received structured interventions through integrated services, including psychoeducation, parent guidance, supportive counselling, and targeted therapies (e.g., CBT, behavioural interventions). Pharmacological management was initiated where indicated. Importantly, prospective follow-up allowed monitoring of treatment response and adherence over time.

Embedding psychiatric care directly within the cleft team normalised mental health input and reduced stigma. Coordination with surgical and speech services appeared to facilitate continuity, supporting the feasibility and acceptability of integrated psychiatric services even in resource-constrained systems.

### Clinical attendance and continuity of care

One of the most important contributions of this study was the linkage of psychiatric morbidity with clinic attendance. Dropout from cleft care was a recognised challenge in LMICs [8]. In the present cohort, despite high feasibility indicators, 93.8% enrollment (210/224), 100% completion of baseline mental health screening, and universal delivery of at least one mental health intervention among children with psychiatric morbidity, longitudinal engagement remained variable, with 28.6% of enrolled children being completely lost to follow-up.

The partial recovery of engagement through hybrid and tele-consultation pathways suggested that flexible follow-up models may enhance acceptability, consistent with evidence demonstrating that telemental health services were effective, acceptable, and improved access across paediatric populations [28].

### Determinants of follow-up continuity in psychiatry-integrated cleft care

In this study, irregular attendance and dropout were associated with socioeconomic vulnerability. Lower socioeconomic status consistently predicted poorer follow-up, aligning with findings from Lynn et al., who reported higher missed appointment rates among families receiving public insurance or need-based financial assistance [29], and Sharif-Askary et al., who identified lower socioeconomic status as a significant predictor of loss to follow-up [30].

Maternal education and parental employment were independently associated with follow-up continuity in our cohort. Although not examined directly by Sharif-Askary et al., these factors were embedded within a socioeconomic index that predicted loss to follow-up, while Lynn et al. reported higher non-attendance among economically vulnerable families. The Nigerian study by Gbolahan et al. similarly identified low parental education and unstable income as contributors to poor follow-up, aligning closely with our findings [31].

Rural residence was associated with poorer follow-up in our cohort, consistent with findings from low-resource settings such as the Nigerian study by Gbolahan et al. [31]. In contrast, Sharif-Askary et al. did not identify rural–urban status or distance as predictors in a high-income setting, suggesting that the impact of geographic barriers on follow-up varied by health system context [30].

Age-related patterns also differed across studies: older age was associated with poorer follow-up continuity in the present cohort, contrasting with Sharif-Askary et al., who identified younger age at last encounter as a predictor of early loss to follow-up [30], and Lynn et al., who found no significant association with age [29].

In contrast, gender, religion, paternal education, cleft type, and hearing impairment were not independently associated with follow-up continuity, suggesting that disengagement in the first year of psychiatry-integrated cleft care was driven more by socioeconomic and caregiver-level vulnerabilities than by clinical or demographic characteristics.

Psychiatric co morbidity was strongly associated with irregular attendance and loss to follow-up in the present cohort. Similar associations were described in cleft-specific literature, where emotional and psychosocial difficulties were linked to avoidance behaviours and reduced engagement with treatment [4, 5, 32], as well as in broader paediatric studies demonstrating that psychiatric morbidity predicted poorer adherence and clinic attendance [33]. These findings reinforced the importance of early identification and integrated mental health support within cleft care pathways.

Together, these findings underscored the central role of socioeconomic, caregiver-level, and contextual factors in shaping follow-up continuity within psychiatry-integrated cleft care.

### Broader determinants of psychosocial outcomes

Early surgical repair and effective speech/hearing rehabilitation remained critical determinants of psychosocial well-being [26, 34]. Speech therapy involvement was significantly associated with poorer follow-up, with higher participation among irregular attendees and dropouts.

Speech therapy involvement showed a significant association with follow-up status, with markedly higher rates among irregular attendees and dropouts compared with regular attendees. While families engaged readily with speech rehabilitation, the cumulative demands of repeated sessions and travel likely contributed to follow-up fatigue and disengagement in resource-limited settings [35].

Hearing impairment, although common, did not differ significantly across follow-up categories, suggesting that when managed within a multidisciplinary framework, it did not independently predict early dropout, consistent with findings reported by Ong and Cormier. However, uncorrected hearing difficulties have been shown to exacerbate communication and emotional challenges [36, 37].

Closer coordination between psychiatry, speech, audiology, and social work services was essential, with embedded psychiatric social workers facilitating appointment adherence and addressing financial and travel barriers contributing to attrition [38]. Collaborative interventions, including counselling around therapy-related fatigue, psychoeducation on gradual progress expectations, and flexible scheduling, may have strengthened adherence and psychosocial outcomes.

### Family feedback and satisfaction

Family feedback demonstrated high acceptability of the psychiatry-integrated cleft care model, with over two-thirds of caregivers expressing satisfaction and citing reduced stigma, caregiver stress relief, and improved understanding of the child’s needs. Dissatisfaction was uncommon and primarily related to logistical barriers such as travel distance or visit frequency rather than objections to mental health involvement.

These findings aligned with established cleft team standards and paediatric collaborative-care literature showing that co-located, multidisciplinary models enhanced family engagement and normalised psychological care by delivering services in a single setting [8, 9, 39].

Importantly, longer-term outcome studies in cleft populations had demonstrated that psychosocial and family-related domains remained vulnerable into adulthood despite satisfactory medical outcomes, underscoring the value of early, integrated mental health input within cleft services [40].

### Contribution of the present study

This study provides one of the first prospective evaluations of integrated psychiatry within a cleft clinic in India. It demonstrates:

1. The high prevalence of psychiatric co morbidities is consistent with international data.
2. A strong prospective association between psychiatric morbidity and clinic dropout.
3. Feasibility and acceptability of psychiatric services embedded in a cleft team, with positive family feedback.
4. There is evidence that integrated interventions can be delivered as part of routine multidisciplinary care.

Together, these findings have important clinical, educational, and policy implications.

### Limitations

Several limitations must be acknowledged:

- Diagnostic variability remains possible despite the use of structured tools, as some conditions (e.g., adjustment disorders) rely on clinical judgement.
- ADHD is only assessed in children ≥ 6 years of age, potentially underestimating its prevalence in younger children.
- Single-centre design may limit generalisability to other settings.
- Attrition bias is possible, as children who drop out may have even higher levels of morbidity not fully captured despite prospective tracking.

### Future directions

Future research should:

- This design can be extended to multicentre, longitudinal studies with larger cohorts.
- Future studies should incorporate validated quality-of-life measures, such as the PedsQL, alongside psychiatric assessments to better capture functional and psychosocial outcomes.
- The impact of integrated psychiatry on long-term surgical, speech, and psychosocial outcomes should be evaluated.
- Emerging longitudinal data demonstrate an increased risk of autism spectrum disorder among children with congenital anomalies [41]. Future integrated cleft–mental health services should therefore incorporate routine ASD screening and longitudinal developmental follow-up to more comprehensively address neurodevelopmental vulnerability.
- Community- and family-level determinants, including parental mental health, stigma, and socioeconomic constraints, should be explored.
- Advocate for the integration of psychiatry into national cleft care protocols.

## Conclusion

This prospective study reinforces psychiatric morbidity as a central determinant of cleft care outcomes, rather than a peripheral concern. By embedding psychiatry within a multidisciplinary cleft team, it is possible to improve adherence, reduce dropout, and address psychosocial vulnerabilities. Families value the model, and its feasibility in a government setting highlights its potential for scale-up in LMIC contexts. Integrating psychiatry into routine cleft protocols should therefore be prioritised to enhance continuity, equity, and long-term well-being.

## Supplementary Information

Additional file 1.

## Data Availability

No datasets were generated or analysed during the current study.
