# Supplementary material for: Integrating mental health assessment and intervention into cleft care: a prospective cohort study from a tertiary clinic in India
Source: Child Adolesc Psychiatry Ment Health. 2026 Feb 13;20:38. doi: 10.1186/s13034-026-01040-5 (PMC13001192; doi:10.1186/s13034-026-01040-5)
Supplement: Supplementary file 1 — Additional file 1. [file 13034_2026_1040_MOESM1_ESM.docx]

**Supplementary Table**: Family feedback form – Integrated cleft clinic

Section A: Overall Experience

1. How satisfied were you with the integrated cleft clinic (all specialists together)?
   - ☐ Very satisfied
   - ☐ Satisfied
   - ☐ Neutral
   - ☐ Dissatisfied
2. Did you feel that psychiatric input was useful for your child and family?
   - ☐ Yes
   - ☐ No
   - ☐ Unsure

Section B: Impact of Psychiatric Support
3. Did counselling/psychoeducation reduce stigma or improve acceptance of your child’s condition within the family/community?

- ☐ Yes
- ☐ No

1. Did psychiatric support help improve adherence to treatment and follow-up visits?
   - ☐ Yes
   - ☐ No
2. Did you find support regarding your child’s school or peer difficulties helpful?
   - ☐ Yes
   - ☐ No
   - ☐ Not applicable
3. Did psychiatric input help reduce your own stress or improve coping as a caregiver?
   - ☐ Yes
   - ☐ No

Section C: Open-ended feedback
7. What aspects of the integrated clinic were most helpful for you and your child?

1. What challenges or difficulties did you face with the integrated clinic?
2. Are there any suggestions for improving the integrated cleft clinic?
